# Case report: Anti-IgLON5 disease and anti-LGI1 encephalitis following COVID-19

**DOI:** 10.3389/fimmu.2023.1195341

**Published:** 2023-06-13

**Authors:** Yanfei Li, Yanjie Jia

**Affiliations:** Department of Neurology, The First Affiliated Hospital of Zhengzhou University, Zhengzhou, Henan, China

**Keywords:** autoimmune encephalitis, IgLON5, LGI1, COVID-19, case report

## Abstract

Anti-IgLON family member 5 (IgLON5) disease is a rare autoimmune encephalitis, characterized by sleep problems, cognitive decline, gait abnormalities, and bulbar dysfunction. Anti–leucine-rich glioma-inactivated 1 (LGI1) autoimmune encephalitis is characterized by cognitive dysfunction, mental disorders, faciobrachial dystonic seizures (FBDS), and hyponatremia. Various studies report that coronavirus disease 2019 (COVID-19) have an effect on the nervous system and induce a wide range of neurological symptoms. Autoimmune encephalitis is one of the neurological complications in severe acute respiratory syndrome coronavirus 2 infection. Until now, autoimmune encephalitis with both anti-IgLON5 and anti-LGI1 receptor antibodies following COVID-19 is rarely reported. The case report described a 40-year-old man who presented with sleep behavior disorder, daytime sleepiness, paramnesia, cognitive decline, FBDS, and anxiety following COVID-19. Anti-IgLON5 and anti-LGI1 receptor antibodies were positive in serum, and anti-LGI1 receptor antibodies were positive in cerebrospinal fluid. The patient presented with typical symptoms of anti-IgLON5 disease such as sleep behavior disorder, obstructive sleep apnea, and daytime sleepiness. Moreover, he presented with FBDS, which is common in anti-LGI1 encephalitis. Therefore, the patient was diagnosed with anti-IgLON5 disease and anti-LGI1 autoimmune encephalitis. The patient turned better after high-dose steroid and mycophenolate mofetil therapy. The case serves to increase the awareness of rare autoimmune encephalitis after COVID-19.

## Introduction

Anti-IgLON5 disease is a rare autoimmune-mediated neurological disorder associated with anti-IgLON5 antibodies against the neuronal cell adhesion protein (1), which was first reported in 2014 as non–rapid eye movement (REM) and REM parasomnia and obstructive sleep apnea (2, 3). The prevalence of anti-IgLON5 disease is about 12 of the 150,000 patients per year, which may be higher because of missed diagnosis and misdiagnosis (4). Because of the low proportions of the disease in total population, reports on the clinical manifestations of anti-IgLON5 disease are still uncommon. The clinical course of anti-IgLON5 disease varies among different patients, such as sleep disorders, gait abnormality, cognitive decline, bulbar symptoms, chorea, and oculomotor problems (5). Patients may develop dysautonomia and life-threatening respiratory problems that need tracheostomy and intensive care support.

Anti–leucine-rich glioma-inactivated 1 (LGI1) encephalitis is an autoimmune encephalitis with antibodies against LGI1, which was first described in 2010. It is the second most frequent style of autoimmune encephalitis, which is featured by limbic encephalitis, cognitive dysfunction, mental disorders, faciobrachial dystonic seizures (FBDS), and hyponatremia (6).

The coronavirus disease 2019 (COVID-19) is an ongoing pandemic caused by the severe acute respiratory syndrome coronavirus 2 (SARS-CoV-2), which has brought a huge public health threat worldwide. Growing evidence demonstrates the association of autoimmune encephalitis with SARS-CoV-2 infection including anti–N-methyl-D-aspartate receptor (NMDAR) antibody encephalitis (7–9), Bickerstaff encephalitis (10), autoimmune encephalitis with anti-NMDAR and anti–glutamic acid decarboxylase (GAD) 65 co-expression (11), and anti–contactin-associated protein–like 2 (CASPR2) encephalitis (12). However, autoimmune encephalitis with both anti-IgLON5 and anti-LGI1 receptor antibodies following COVID-19 has not been reported before. We present a case of a 40-year-old man who was diagnosed with anti-IgLON5 disease and anti-LGI1 autoimmune encephalitis following COVID-19, which expand the knowledge about the clinical spectrum of encephalitis after COVID-19.

## Case description

A 40-year-old man started to have complaint of fatigue and fever in December 2022. SARS-CoV-2 polymerase chain reaction was positive in throat swab. After approximately 15 days, he gradually developed anxiety and paroxysmal dizziness and then presented with left limbs and mouth involuntary movement, sleep behavior disorders (talking, grabbing, and kicking during sleep), daytime sleepiness, paramnesia, and cognitive decline with predominant short-term memory loss.

The patient had no prior medical history. His neurological examination was normal apart from memory decline and paroxysmal involuntary movement. His mini-mental state examination (MMSE) scores were 26 [reference range (27–30)] and Montreal cognitive assessment (MoCA) scores were 23 [reference range (26–30)].

The serum sodium level was 133 mmol/L (normal range, 135–155 mmol/L). Thyroid gland function tests showed free thyroxine level of 19.82 pmol/L (normal range, 7.9–18.4 pmol/L). Tumor markers showed an increased carcinoembryonic antigen level of 5.06 ng/ml (0–5 ng/mL) and cytokeratin 19 fragment level of 3.91 ng/ml (0.1–3.3 ng/mL). Other laboratory tests were normal, including urinalysis test, blood routine index, liver function, renal function, glucose, erythrocyte sedimentation rate, glycosylated hemoglobinscoagulation profiles, blood lipid, antinuclear antibodies, double strand DNA, and antineutrophil cytoplasmic antibodies. Infections of hepatitis B virus, hepatitis C virus, syphilis, and human immunodeficiency virus were negative.

**Figure 1:**
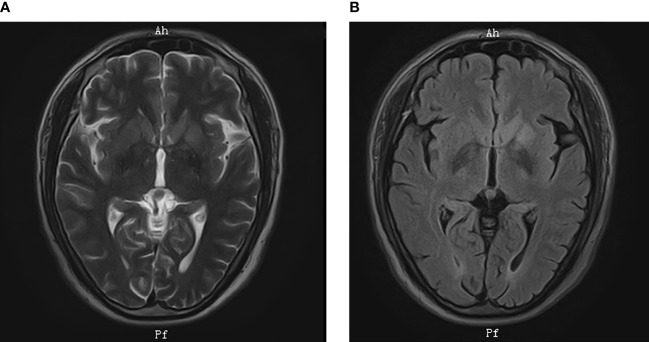
Brain MRI. MRI showed high signal intensity in the left basal ganglia on **(A)** T2-weighted images; **(B)** FLAIR sequences.

**Figure 2:**
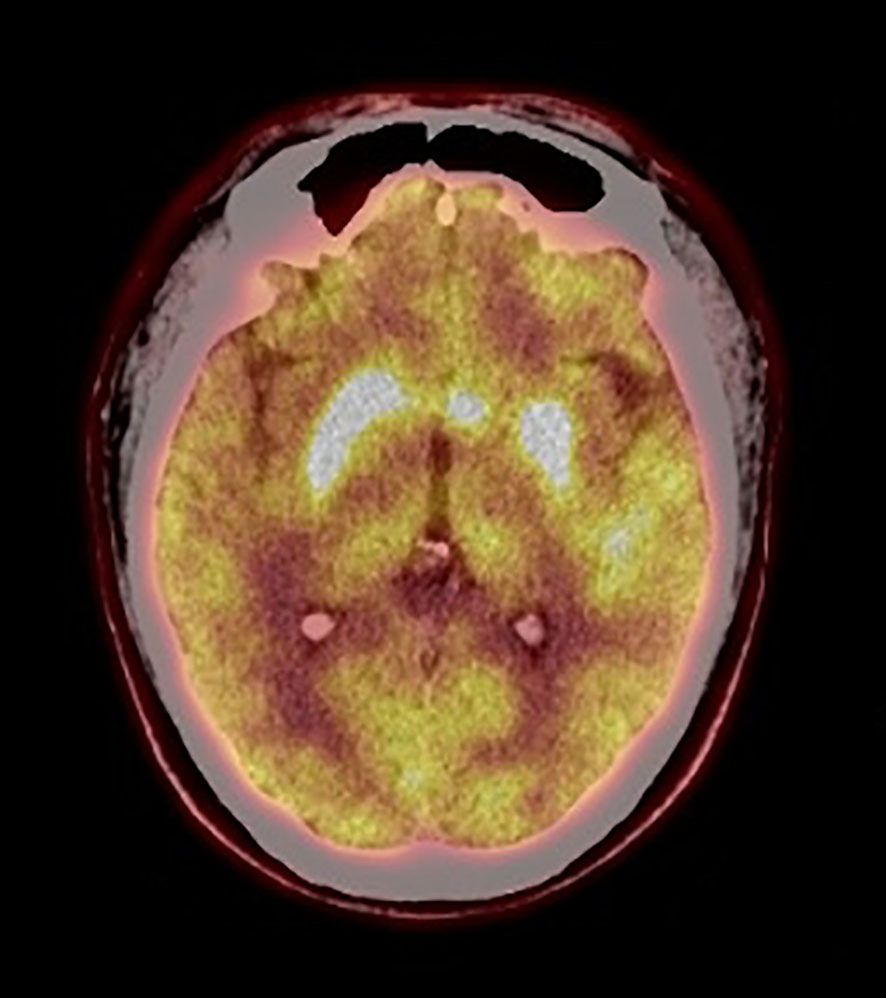
PET-CT imaging. PET-CT showed hypermetabolism level of bilateral caudate nucleus, putamen, and left temporal lobes.

Electroencephalogram was normal. Polysomnography revealed moderate chronic obstructive sleep apnea (apnea hypopnea index, 28/h). Electrocardiography, computed tomography (CT) of chest, and nasopharynx were normal. Abdominal color Doppler ultrasonography and cardiac color Doppler ultrasonography were normal. Brain magnetic resonance imaging showed high signal intensity on T2-weighted images and on fluid-attenuated inversion recovery (FLAIR) sequences in the left basal ganglia with no enhancement (**Figure 1**). Positron emission tomography (PET)–CT imaging showed the hypermetabolism level of bilateral caudate nucleus, putamen, and left temporal lobes (**Figure 2**).

A lumbar puncture was also performed. The cerebrospinal fluid (CSF) pressure was 145 mmH_2_O. The glucose, oligoclonal bands of immunoglobin G (IgG), and the number of cells were found to be normal. In addition, the protein level was 680.9 mg/L (normal range, 150–450 mg/L). Next-generation sequencing for pathogen detection in CSF was negative. Amyloidβ42 (Aβ42) was 656.72 pg/ml (negative: ≥888.1pg/ml; probable positive: 650.1–888.0 pg/ml; positive: ≤650.0 pg/ml). Phosphorylated tau protein 181 was normal. Total tau protein was 440.52 pg/ml (negative: ≤378.0 pg/ml; probable positive: 378.1–398.9 pg/ml; positive: ≥399.0 pg/ml). An assay panel of autoimmune encephalitis including autoantibodies against NMDAR, LGI-1, IgLON5, contactin-associated protein–like 2 (CASPR2), metabotropic g-aminobutyric acid type B (GABAB) receptors, GAD, alpha-amino-3-hydroxy-5-methyl-4-isoxazolepropionic acid receptor, metabolic glutamate receptor 5, dopamine 2 receptor, dipeptidyl-peptidase–like protein 6, and glycine receptor were implied (cell-based assay). He was positive for anti-LGI1 antibodies (1:1,000 in the serum and 1:100 in the CSF) and for anti-IgLON5 antibodies (1:100 in the serum).

The patient was diagnosed with anti-IgLON5 disease and anti-LGI1 autoimmune encephalitis finally. Then, the patient proceeded to treatment with methylprednisolone pulse therapy (500 mg per day for 3 days, 250 mg per day for 3 days, 120 mg per day for 3 days, 80 mg per day for 3 days, and then 60 mg per day orally with a gradual reduction). The patient received mycophenolate mofetil (0.5 g, twice daily) treatment when the oral steroids started. After 2 weeks of immunotherapy, the patient’s symptoms of cognitive function, FBDS, and sleep behavior disorder were obviously alleviated. His MoCA score was 24 and MMSE score was 30. The autoantibodies against LGI-1 and IgLON5 in serum were 1:100 and 1:10, respectively. Then, he was discharged (**Supplementary Table 1**).

## Discussion

We here described a patient presented with anxiety, paroxysmal dizziness, FBDS, sleep behavior disorders, daytime sleepiness, paramnesia, and cognitive decline following COVID-19. Anti-IgLON5 and anti-LGI1 antibodies were positive in serum, and anti-LGI1 receptor antibodies were positive in CSF. The patient presented with typical symptoms of anti-IgLON5 disease such as sleep behavior disorder and daytime sleepiness. In addition, polysomnography revealed moderate chronic obstructive sleep apnea. Moreover, he presented with FBDS, which is common in anti-LGI1 encephalitis. Although there is still no definite diagnostic criteria for anti-IgLON5 disease, positive antibodies in CSF or serum are a relatively reliable diagnostic implication. According to the diagnostic criteria of autoimmune encephalitis reported by Graus et al. (13), the patient was finally diagnosed with anti-IgLON5 disease with anti-LGI1 autoimmune encephalitis.

Double-positive antibodies of anti-LGI1 and other autoimmune antibodies have been reported in the previous studies. Zhang et al., Lai et al., and Klein et al. have reported 10 patients whose anti-CASPR2 and anti-LGI1 antibodies were both positive (14–16). Kunchok et al. collected 42,032 patients in the Mayo Clinic Neuroimmunology Laboratory between January 2018 and December 2019 for autoimmune encephalitis antibodies. Among these patients, 13 cases presented with double-positive antibodies of anti-LGI1 and anti-CASPR2, one case presented with anti-LGI1 and anti-NMDAR, and one case presented with anti-LGI1 and anti–myelin oligodendrocyte glycoprotein (MOG) antibodies (17).

Until now, double-positive antibodies of IgLON5 and other autoimmune antibodies are rarely reported, and there is a lack of detailed description of clinical symptoms and imaging results. Chen et al. recruited 13 patients with positive IgLON5 antibodies in serum and/or CSF and found that coexisting neural autoantibodies were identified in two patients (18), which were anti-LGI1 and anti-MOG, respectively. Chung et al. reported a patient with positive anti-IgLON5 antibodies and anti-GABAB receptor antibodies in serum and positive anti-IgLON5 antibodies in CSF (19). Honorat et al. summarized neurological symptoms and outcomes in 20 patients with IgLON5 antibody and reported that two patients had coexisting antibodies detected in serum (LGI-1 antibodies and GAD65 antibodies) (20). Different antineuronal antibodies are associated with distinct subtypes of autoimmune encephalitis. When encountered with overlapping of multiple antibodies, it is important to identify coexisting antibodies or culprit antibodies. The case reported by Chung presented with sleep disorders, gait abnormality, cognitive decline, hallucinations, depression, and dysarthria, whose clinical manifestations are classical symptoms of anti-IgLON5 disease. However, he did not exhibit typical characters of anti–GABAB receptor–associated autoimmune encephalitis such as epilepsy. The authors speculated the lack of anti-GABAB antibodies in CSF may be one reason why the clinical syndrome does not show typical characteristics of GABAB receptor encephalitis. Moreover, we also suppose anti-GABAB receptor may be coexisting antibodies other than culprit antibodies. In the present case, the patient showed both sleep disorders and FBDS, which were typical symptoms of anti-IgLON5 disease and anti-LGI1 autoimmune encephalitis, respectively. In addition, the patient showed obvious clinical improvement with decline of antibodies titers after treatment. Therefore, we consider double positive antibodies as “culprit” antibodies.

Nabizadeh et al. reviewed different cases of autoimmune encephalitis following SARS-CoV-2 infection. They summarized 19 cases diagnosed with autoimmune encephalitis among the studies. The most common seen cases were limbic encephalitis (37%), and the other cases were anti-NMDA receptor encephalitis (26%), new-onset refractory status epilepticus (11%), steroid-responsive encephalitis (5%), and unknown type of autoimmune encephalitis (21%), respectively (21). To the best of our knowledge, this is the first case of anti-IgLON5 disease and anti-LGI1 encephalitis following COVID-19. Autoimmune encephalitis is mainly presented several days after respiratory symptoms, which suggest that COVID-19 infection may play a trigger role in autoimmune disease of the central nervous system (CNS). The underlying mechanisms of autoimmune encephalitis after COVID-19 infection have not yet been elucidated. The most possible mechanism is molecular mimicry in response to the COVID-19 infection, which induce activation of autoimmune and then host antibodies recognize self-antigens as foreign and attack CNS. Another possible mechanism is the release of cytokine mediated neuroinflammation after SARS-CoV-2 infection, which are called “cytokine storm”. Patients with SARS-CoV-2 infection express high concentrations of proinflammatory biomarkers, such as d-dimer, interleukin-6 (IL-6), and high-sensitive C-reactive protein (7), which may result in inflammatory damage of CNS. The third mechanism is direct invasion of the virus into the CNS. However, among patients who presented autoimmune encephalitis after COVID-19, only few are positive cases for IgG and IgM in CSF for SARS-CoV-2 (22, 23). The present case showed a negative result of next-generation sequencing for pathogen detection in CSF. It was speculated that the presence of the virus in CSF may be transient.

To draw a conclusion, we reported a case presented with anti-IgLON5 disease and anti-LGI1 encephalitis following COVID-19, which serve to increase the awareness of rare autoimmune encephalitis after COVID-19. This awareness is important as timely diagnosis, and treatment is necessary to achieve a favorable prognosis.

## Patient perspective

The patient and his wife were satisfied with the improvement in his condition after receiving immunotherapy. He agreed with us to share his case after understanding that his condition is quite rare.

## Data availability statement

The raw data supporting the conclusions of this article will be made available by the authors, without undue reservation.

## Ethics statement

The studies involving human participants were reviewed and approved by Ethics committee of First Affiliated Hospital of Zhengzhou University. The patients/participants provided their written informed consent to participate in this study. Written informed consent was obtained from the individual(s) for the publication of any potentially identifiable images or data included in this article.

## Author contributions

YL was responsible for acquisition and interpretation of the autoimmune encephalitis panel and wrote original draft. YJ was responsible for conceptualization, supervision, and revision of the manuscript. All authors contributed to the article and approved the submitted version.

## References

[1] Ni Y Feng Y Shen D Chen M Zhu X Zhou Q. Anti-IgLON5 antibodies cause progressive behavioral and neuropathological changes in mice. J Neuroinflamm (2022) 19(1):140. doi: 10.1186/s12974-022-02520-z PMC9188070 35690819

[2] Balint B Bhatia KP. Friend or foe? IgLON5 antibodies in a novel tauopathy with prominent sleep movement disorder, ataxia, and chorea. Mov Disord (2014) 29(8):989. doi: 10.1002/mds.25926 24865449

[3] Sabater L Gaig C Gelpi E Bataller L Lewerenz J Torres-Vega E. A novel non-rapid-eye movement and rapid-eye-movement parasomnia with sleep breathing disorder associated with antibodies to IgLON5: a case series, characterisation of the antigen, and post-mortem study. Lancet Neurol (2014) 13(6):575–86. doi: 10.1016/S1474-4422(14)70051-1 PMC4104022 24703753

[4] Madetko N Marzec W Kowalska A Przewodowska D Alster P Koziorowski D. Anti-IgLON5 disease - the current state of knowledge and further perspectives. Front Immunol (2022) 13:852215. doi: 10.3389/fimmu.2022.852215 35300333 PMC8921982

[5] Gaig C Graus F Compta Y Högl B Bataller L Brüggemann N. Clinical manifestations of the anti-IgLON5 disease. Neurology (2017) 88(18):1736–43. doi: 10.1212/WNL.0000000000003887 PMC5409845 28381508

[6] Otiniano-Sifuentes R Cuba Antezana A de la Cruz Ramirez WF Pacheco-Barrios K Segura Chavez DA. Case report: anti-LGI1 limbic encephalitis associated with anti-thyroid autoantibodies. Front Neurol (2021) 11:620483. doi: 10.3389/fneur.2020.620483 33519701 PMC7843919

[7] Payus AO Jeffree MS Ohn MH Tan HJ Ibrahim A Chia YK. Immune-mediated neurological syndrome in SARS-CoV-2 infection: a review of literature on autoimmune encephalitis in COVID-19. Neurol Sci (2022) 43(3):1533–47. doi: 10.1007/s10072-021-05785-z PMC8635316 34853897

[8] Sarigecili E Arslan I Ucar HK Celik U. Pediatric anti-NMDA receptor encephalitis associated with COVID-19. Childs Nerv Syst (2021) 37(12):3919–22. doi: 10.1007/s00381-021-05155-2 PMC8045445 33852058

[9] Monti G Giovannini G Marudi A Bedin R Melegari A Simone AM. Anti-NMDA receptor encephalitis presenting as new onset refractory status epilepticus in COVID-19. Seizure (2020) 81:18–20. doi: 10.1016/j.seizure.2020.07.006 32688169 PMC7362825

[10] Llorente Ayuso L Torres Rubio P Beijinho do Rosário RF Giganto Arroyo ML Sierra-Hidalgo F. Bickerstaff encephalitis after COVID-19. J Neurol (2021) 268(6):2035–7. doi: 10.1007/s00415-020-10201-1 PMC7471525 32880723

[11] Valadez-Calderon J Ordinola Navarro A Rodriguez-Chavez E Vera-Lastra O. Co-Expression of anti-NMDAR and anti-GAD65 antibodies. a case of autoimmune encephalitis in a post-COVID-19 patient. Neurologia (2022) 37(6):503–4. doi: 10.1016/j.nrl.2021.09.003 PMC8556136 34744237

[12] Subramanyam M Gupta SB Chamraj S. Caspr2 autoimmune encephalitis with COVID-19 infection. J Assoc Physicians India (2022) 70(6):11–2. 35702850

[13] Graus F Titulaer MJ Balu R Benseler S Bien CG Cellucci T. A clinical approach to diagnosis of autoimmune encephalitis. Lancet Neurol (2016) 15(4):391–404. doi: 10.1016/S1474-4422(15)00401-9 26906964 PMC5066574

[14] Zhang L Lu Q Guan HZ Mei JH Ren HT Liu MS. A Chinese female morvan patient with LGI1 and CASPR2 antibodies: a case report. BMC Neurol (2016) 16:37. doi: 10.1186/s12883-016-0555-x 26983964 PMC4793739

[15] Lai M Huijbers MG Lancaster E Graus F Bataller L Balice-Gordon R. Investigation of LGI1 as the antigen in limbic encephalitis previously attributed to potassium channels: a case series. Lancet Neurol (2010) 9(8):776–85. doi: 10.1016/S1474-4422(10)70137-X PMC3086669 20580615

[16] Klein CJ Lennon VA Aston PA McKeon A O'Toole O Quek A. Insights from LGI1 and CASPR2 potassium channel complex autoantibody subtyping. JAMA Neurol (2013) 70(2):229–34. doi: 10.1001/jamaneurol.2013.592 PMC3895328 23407760

[17] Kunchok A McKeon A Zekeridou A Flanagan EP Dubey D Lennon VA. Autoimmune/Paraneoplastic encephalitis antibody biomarkers: frequency, age, and sex associations. Mayo Clin Proc (2022) 97(3):547–59. doi: 10.1016/j.mayocp.2021.07.023 34955239

[18] Ni Y Shen D Zhang Y Song Y Gao Y Zhou Q. Expanding the clinical spectrum of anti-IgLON5 disease: a multicenter retrospective study. Eur J Neurol (2022) 29(1):267–76. doi: 10.1111/ene.15117 34543501

[19] Chung HY Wickel J Voss A Ceanga M Sell J Witte OW. Autoimmune encephalitis with anti-IgLON5 and anti-GABAB-receptor antibodies: a case report. Med (Baltimore) (2019) 98(20):e15706. doi: 10.1097/MD.0000000000015706 PMC6531245 31096519

[20] Honorat JA Komorowski L Josephs KA Fechner K St Louis EK Hinson SR. IgLON5 antibody: neurological accompaniments and outcomes in 20 patients. Neurol Neuroimmunol Neuroinflamm (2017) 4(5):e385. doi: 10.1212/NXI.0000000000000385 28761904 PMC5515599

[21] Nabizadeh F Balabandian M Sodeifian F Rezaei N Rostami MR Naser Moghadasi A. Autoimmune encephalitis associated with COVID-19: a systematic review. Mult Scler Relat Disord (2022) 62:103795. doi: 10.1016/j.msard.2022.103795 35472834 PMC8983076

[22] Ayatollahi P Tarazi A Wennberg R. Possible autoimmune encephalitis with claustrum sign in case of acute SARS-CoV-2 infection. Can J Neurol Sci (2021) 48(3):430–2. doi: 10.1017/cjn.2020.209 PMC7653487 32938510

[23] Zambreanu L Lightbody S Bhandari M Hoskote C Kandil H Houlihan CF. A case of limbic encephalitis associated with asymptomatic COVID-19 infection. J Neurol Neurosurg Psychiatry (2020) 91(11):1229–30. doi: 10.1136/jnnp-2020-323839 32661082

