# Supplementary material for: Case report: Anti-IgLON5 disease and anti-LGI1 encephalitis following COVID-19
Source: Front Immunol. 2023 Jun 13;14:1195341. doi: 10.3389/fimmu.2023.1195341 (PMC10293611; doi:10.3389/fimmu.2023.1195341)
Supplement: Supplementary file 1 [file Table_1.docx]

Supplementary Table 1 Timeline with relevant data from the patient’s course of clinical symptoms and treatment.

| Date | Symptoms, treatment and outcome |
| --- | --- |
| 2022.12.15 | Fever and SARS-CoV-2 PCR was positive. |
| 2023.1.1 | Dizziness |
| 2023.1.15 | FBDS |
| 2023.1.20 | Sleep behavior disorders, depression |
| 2023.2.6 | Cognitive decline, daytime sleepiness, paramnesia |
| 2023.2.12 | Anti-LGI1 antibodies were detected in serum (1:1000+) and CSF (1:100+), anti-IgLON5 antibodies were detected in serum (1:100+),  23/30 on MoCA and 26/30 on MMSE |
| 2023.2.12 | Start of high-dose steroid therapy |
| 2023.2.23 | Mycophenolate mofetil (0.5g, twice daily) was added. FBDS, cognitive decline, daytime sleepiness, paramnesia and psychiatric symptoms were alleviated. |
| 2023.2.24 | MoCA score was 24 and MMSE score was 30. The autoantibodies against LGI-1 and IgLON5 in serum were 1:100 and 1:10 respectively. |

Note: PCR, polymerase chain reaction; FBDS, faciobrachial dystonic seizures; anti-LGI1, anti-leucine-rich glioma-inactivated 1; CSF, Cerebrospinal fluid; MoCA, Montreal Cognitive Assessment; MMSE, Mini-Mental State Examination.
